# Clinicopathologic Profile of Breast Cancer in Germline ATM and CHEK2 Mutation Carriers

**DOI:** 10.3390/genes12050616

**Published:** 2021-04-21

**Authors:** Angela Toss, Elena Tenedini, Claudia Piombino, Marta Venturelli, Isabella Marchi, Elisa Gasparini, Elena Barbieri, Elisabetta Razzaboni, Federica Domati, Federica Caggia, Giovanni Grandi, Francesca Combi, Giovanni Tazzioli, Massimo Dominici, Enrico Tagliafico, Laura Cortesi

**Affiliations:** 1 Department of Oncology and Hematology, Azienda Ospedaliero Universitaria di Modena, 41124 Modena, Italy; claudia.piombino@outlook.com (C.P.); martaventurelli@msn.com (M.V.); marchi.isabella@policlinico.mo.it (I.M.); barbieri.elena@aou.mo.it (E.B.); federica.domati@unimore.it (F.D.); federica.caggia@unimore.it (F.C.); massimo.dominici@unimore.it (M.D.); hbc@unimore.it (L.C.); 2 Department of Surgery, Medicine, Dentistry and Morphological Sciences with Transplant Surgery, Oncology and Regenerative Medicine Relevance, University of Modena and Reggio Emilia, 41124 Modena, Italy; 3 Department of Medical and Surgical Sciences, University of Modena and Reggio Emilia, 41124 Modena, Italy; tenedini.elena@aou.mo.it (E.T.); giovanni.grandi@unimore.it (G.G.); giovanni.tazzioli@unimore.it (G.T.); enrico.tagliafico@unimore.it (E.T.); 4 Department of Oncology, Arcispedale S. Maria Nuova IRCCS, 42123 Reggio Emilia, Italy; gaspariniel@ausl.re.it; 5 Hospital Psychology Service, Azienda Ospedaliero Universitaria di Modena, 41124 Modena, Italy; razzaboni.elisabetta@aou.mo.it; 6 Obstetrics and Gynecology Unit, Department of Obstetrics, Gynecology and Pediatrics, Azienda Ospedaliero Universitaria di Modena, 41124 Modena, Italy; 7 Department of Biomedical, Metabolic and Neural Sciences, International Doctorate School in Clinical and Experimental Medicine, University of Modena and Reggio Emilia, 41124 Modena, Italy; francesca.combi@unimore.it; 8 Division of Breast Surgical Oncology, Department of Medical and Surgical, Maternal-Infantile and Adult Sciences, Azienda Ospedaliero Universitaria di Modena, 41124 Modena, Italy; 9 Diagnostic Hematology and Clinical Genomics Unit, Department of Laboratory Medicine and Pathology, Azienda Ospedaliero Universitaria di Modena, 41124 Modena, Italy; 10 Center for Genome Research, University of Modena and Reggio Emilia, 41124 Modena, Italy

**Keywords:** breast cancer, *ATM*, *CHEK2*, genetic testing, bilateral tumor, mastectomy

## Abstract

The most common breast cancer (BC) susceptibility genes beyond *BRCA1/2* are *ATM* and *CHEK2*. For the purpose of exploring the clinicopathologic characteristics of BC developed by *ATM* or *CHEK2* mutation carriers, we reviewed the archive of our Family Cancer Clinic. Since 2018, 1185 multi-gene panel tests have been performed. Nineteen *ATM* and 17 *CHEK2* mutation carriers affected by 46 different BCs were identified. A high rate of bilateral tumors was observed in *ATM* (26.3%) and *CHEK2* mutation carriers (41.2%). While 64.3% of *CHEK2* tumors were luminal A-like, 56.2% of *ATM* tumors were luminal B-like/HER2-negative. Moreover, 21.4% of *CHEK2*-related invasive tumors showed a lobular histotype. About a quarter of all *ATM*-related BCs and a third of *CHEK2* BCs were in situ carcinomas and more than half of *ATM* and *CHEK2*-related BCs were diagnosed at stage I-II. Finally, 63.2% of *ATM* mutation carriers and 64.7% of *CHEK2* mutation carriers presented a positive BC family history. The biological and clinical characteristics of *ATM* and *CHEK2*-related tumors may help improve diagnosis, prognostication and targeted therapeutic approaches. Contralateral mastectomy should be considered and discussed with *ATM* and *CHEK2* mutation carriers at the first diagnosis of BC.

## 1. Introduction

The recent introduction of multigene panel testing for mutations associated with breast and/or ovarian cancer has raised new challenges in the management of both individuals at increased cancer risk and cancer patients. In addition to the known high-penetrance *BRCA1/2* mutations, pathogenic variants in other high/moderate-penetrance genes can increase the risk of breast and/or ovarian cancer. Nevertheless, while providing risk assessment, their clinical utility in terms of primary and secondary prevention, prognostication and treatment modalities are still uncertain [1].

**Figure 1:**
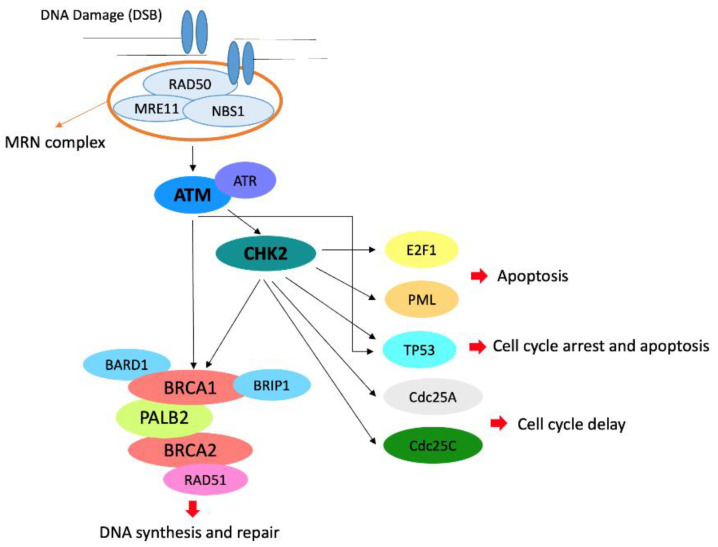
Role of ATM and CHK2 in the pathways of cell cycle arrest, apoptosis, DNA repair and mitosis. In particular, the MRN complex resects DNA at the double-strand break (DSB) and recruits ATM that phosphorylates CHK2 and recruits the BRCA complex.

The most common non-*BRCA* pathogenic or likely pathogenic variants were found in *ATM* and *CHEK2* genes [2]. In particular, the mutation frequency for *ATM* is 0.78% and for *CHEK2* 1.08% in unselected breast cancer patients, whereas the prevalence in unaffected women is 0.41% for *ATM* and 0.42% for *CHEK2* [3]. Both *ATM* and *CHEK2* are considered as moderate-penetrance genes and are involved in DNA-double strand break repair mechanisms [4]. In particular, the ATM protein kinase is a critical intermediary in a number of cellular responses to ionizing irradiation and possibly other stresses. In addition, its dysfunction results in abnormal checkpoint responses in multiple phases of the cell cycle [5]. After DNA damage, ATM and DNA-dependent (DNA-PK) protein kinase activate CHK2, which in turn phosphorylates a number of downstream substrates involved in various cellular processes, including cell cycle arrest, apoptosis, DNA repair and mitosis [6] (Figure 1).

Individuals carrying heterozygous pathogenic variants in *ATM* present a 33% cumulative lifetime risk for BC by 80 years of age [7], whereas certain variants in the *CHEK2* gene are associated with increased BC risk, with a cumulative lifetime risk ranging from 28% to 37% depending on family history [8,9]. Due to this increased risk, for both *ATM* and *CHEK2* carriers, mammogram with consideration of breast MRI is recommended yearly from 40 years of age according to the current National Comprehensive Cancer Network (NCCN) guidelines [10]. Although only insufficient data are available, furthermore, bilateral risk-reducing mastectomy may be considered, based on family history [10]. Additionally, *ATM* heterozygous pathogenic variants have also been described in some cases of familial ovarian [11], pancreatic [12], and prostate cancer [13], whereas pathogenic *CHEK2* variants were also associated to an increased risk of other malignancies including colon, prostate, kidney, bladder and thyroid cancers, according to specific mutations (frameshift or missense substitutions) [14].

So far, only a few small-sample studies investigated whether BC developed by *ATM* or *CHEK2* mutation carriers includes distinct histopathological features and clinical outcomes from sporadic BC and *BRCA1/2* associated tumors. Renault et al. [15] showed that most *ATM*-associated tumors are luminal B or luminal B/HER2+ tumors. Nizic-Kos et al. [16] reported that the majority of patients with *CHEK2* pathogenic or likely pathogenic variants develop luminal A or luminal B BC subtypes. In a recent retrospective case-control study, finally, Bergstrom and colleagues [17] found that BC patients with germline pathogenic variants of *ATM*, *CHEK2*, or *PALB2* have an increased family history of breast cancer, tumor size >2.0 cm at the time of diagnosis, and potentially an increased risk of recurrence compared to mutation-negative patients. However, lymph nodes, nuclear grade, histology, Ki-67 proliferation and receptor status were not different from sporadic tumors.

The aim of our study was to explore whether the presence of *ATM* or *CHEK2* pathogenic or likely pathogenic germline variants in BC patients is associated with specific clinicopathologic characteristics and prognostic features at our institution.

## 2. Materials and Methods

### 2.1. Study Population and Design

The Modena Family Cancer Clinic (MFCC), located in the Emilia Romagna region (Northern Italy), offers genetic counseling to individuals with a personal or family history of BC and/or ovarian cancer (OC) in accordance with regional criteria for *BRCA* genetic testing [18]. Since the 8 January 2018, counseling has also been given to all patients affected by pancreatic cancer (PC) following Olaparib approval as a first-line maintenance treatment (Table 1). During pre-test counseling, family and personal histories of cancer are collected. At the same time, a family pedigree is drawn including third-degree relatives on both maternal and paternal sides. In particular, healthy women with BC and/or OC family history are referred to the MFCC by general practitioners or radiologists that perform population-based screening mammography. On the other hand, BC, OC and PC patients are referred to the MFCC by oncologists, radiologists, surgeons or gynecologists. Eligible individuals can undergo genetic testing. Then, in case of a positive result, the option of searching for specific pathogenic or likely pathogenic variant can be provided to other family members, in order to access risk-reducing surgery [19], chemoprevention studies [20] or more intensive surveillance programs [21,22]. After post-test counseling, finally, a copy of all patient documents and reports is stored in the MFCC archive.

Between 1998 and 2017, the MFCC offered *BRCA1/2* genetic testing to BC and/or OC patients, first according to the Modena Criteria for genetic testing [23,24] and subsequently, according to the criteria recommended by the Emilia Romagna region [18]. On the 8 January 2018, the Clinical Genomics Laboratory of the MFCC started to provide a Next Generation Sequencing (NGS) multigene panel testing to all new patients who met the Regional Criteria for *BRCA* genetic testing and all PC patients. Furthermore, patients who tested negative for BRCA genes in the previous years were recalled to undergo the new multi-gene panel test. Clinical and pathologic characteristics of BC patients testing positive for variants classified as pathogenic or likely pathogenic in the *ATM* or *CHEK2* genes were then collected. These included age at first diagnosis, histotype, immunohistochemical profile of invasive carcinomas, clinical stage at diagnosis, type of breast and axillary surgery, radiotherapy, chemotherapy and rate of recurrence.

Estrogen Receptor (ER), Progesterone Receptor (PgR) and Human Epidermal growth factor Receptor 2 (HER2) expression were determined according to national pathology guidelines, which closely adhere to international standards [25,26]. According to the ESMO Clinical Practice Guidelines [27], for the purpose of prognostication and treatment decision making, tumors should be grouped into surrogate intrinsic subtypes, defined by routine histology and IHC data. In our study, in line with the 2013 St Gallen Consensus Conference [28] and local laboratory values, luminal A-like tumors have been defined as ER-positive, PgR ≥20%, HER2-negative and Ki67 <20%. On the other hand, luminal B-like tumors are characterized by ER-positive, and either Ki67 high (≥20%) or PgR low (<20%) or HER2-positive.

All subjects gave their informed consent for inclusion before they participated in the study. The study was conducted in accordance with the Declaration of Helsinki, and the protocol was approved by the Ethics Committee of the Area Vasta Emilia Nord (Project identification code 125/2021/OSS*/AOUMO, Modena, Italy).

### 2.2. Procedures for Multi-Gene Panel Testing

Peripheral blood samples (PB) were collected into EDTA tubes, in accordance with the current revision of the Helsinki Declaration, and genomic DNA was extracted with the DNA Midi Kit via QIASymphony platform (Qiagen, Hilden, Germany); nucleic acid quantity/quality were checked by Qubit dsDNA High Sensitivity kit and Nanodrop (Thermo Scientific, Waltham, MA, USA).

Sequencing libraries were prepared using the CE-IVD SOPHiA HCS v1.1 kit, exclusively through the automated procedure implemented on the STARlet platform (Hamilton, https://www.hamiltoncompany.com/press-releases/application-note-automation-of-the-hereditary-cancer-solution-hcs-by-sophia-genetics-on-a-starlet#top, accessed on 20 April 2021). Individual library quantification was performed via fluorometric quantitation by Qubit dsDNA High Sensitivity kit (Thermo Scientific, Waltham, Massachusetts, USA) and quality control analysing the profile of each sample via capillary electrophoresis with Bioanalyzer DNA 1000 (Agilent Technologies, Santa Clara, CA, USA). Samples were run onto a 600-cycle format V3 flow-cell and sequenced via Illumina MiSeq DX platform according to their own and SOPHiA GENETICS’ (Lausanne, Switzerland; Boston, MA, USA) protocols.

The SOPHiA HCS allows for the enrichment of coding and splicing regions of 26 genes (APC, ATM, BARD1, BRCA1, BRCA2, BRIP1, CDH1, CHEK2, EPCAM, FAM175A, MLH1, MRE11A, MSH2, MSH6, MUTYH, NBN, PALB2, PIK3CA, PMS2, PTEN, RAD50, RAD51C, RAD51D, STK11, TP53, XRCC2) and the pseudogene PMS2CL. This is known to be associated with increased risk for cancer syndromes. The sequencing data were simultaneously processed for single nucleotide variants (SNVs), indels and copy number variations (CNVs) using the SOPHiA DDM software (DDM) updated to the last available version at the time of sequencing. In accordance with local and international guidelines as well as with the patients’ informed consent, data analysis and variant interpretation were limited to the following actionable gene set: APC, ATM, BARD1, BRCA1, BRCA2, BRIP1, CDH1, CHEK2, EPCAM, MLH1, MSH2, MSH6, MUTYH, NBN, PALB2, PMS2, PTEN, RAD50, RAD51C, RAD51D, STK11, TP53. Genetic variant annotations were also integrated with data from in literature and open source bioinformatics tools customized and validated in the laboratory (Annovar [29] and Variant Effect Predictor (VEP) [30]), and through consultation of specific databases: Leiden Open source Variation Database (https://grenada.lumc.nl/LOVD2/mendelian_genes/home.php? accessed on 20 April 2021), ClinVar (http://www.ncbi.nlm.nih.gov/clinvar/ accessed on 20 April 2021), 1000 Genomes Project (http://www.1000genomes.org/data accessed on 20 April 2021), ExAC (http://exac.broadinstitute.org/ accessed on 20 April 2021), dbSNP (http://www.ncbi.nlm.nih.gov/projects/SNP/ accessed on 20 April 2021), The Genome Aggregation Database (http://gnomad.broadinstitute.org/ accessed on 20 April 2021), BRCA Share (http://www.umd.be/BRCA1/ http://www.umd.be/BRCA2/ accessed on 20 April 2021). Variants were reported using the international standard HGVS nomenclature and a classification into 5 classes (Pathogenic, Likely Pathogenic, Variant of Uncertain Significance, Likely Benign and Benign), according to the American College of Medical Genetics and Genomics (ACMG, Bethesda, MD, USA) criteria [31].

All gene variants or CNVs interpreted as Pathogenic or Likely Pathogenic were confirmed by Sanger sequencing performed with predesigned primers (the BigDye Direct Cycle Sequencing Kit), analyzed through the Applied Biosystems 3500xL Dx Genetic Analyzer platform and SeqScape3 software (Thermo Scientific), or by MLPA (MRC-Holland, Amsterdam, The Netherlands) and examined through the Coffalyser.Net software (MRC-Holland) updated to the latest available version.

## 3. Results

### 3.1. Overall Population

**Figure 2:**
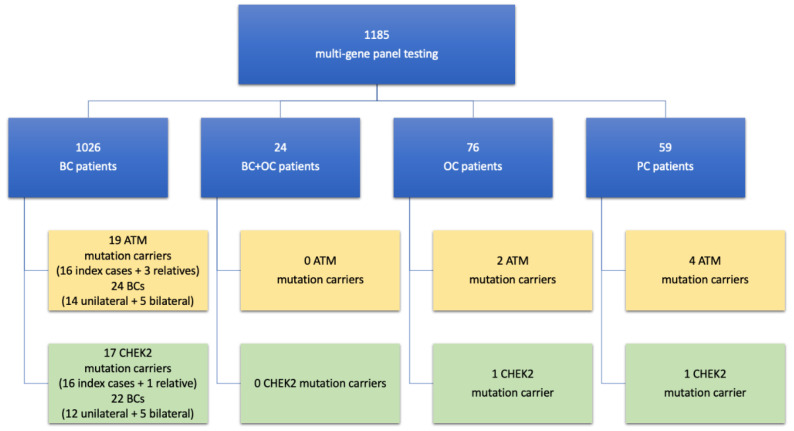
Study flow-chart.

Since the 8 January 2018, 1185 multi-gene panel tests have been performed. Of these tests, 1026 were performed on BC patients (422 of these on those who previously tested negative for BRCA genes). In addition, 24 were performed on BC and OC patients, with 11 of these previously testing negative for BRCA genes. Moreover, 76 tests were conducted on OC patients, 48 of these previously testing negative for BRCA genes, and 59 on PC patients (never tested before). Overall, *ATM* pathogenic or likely pathogenic germline variants were found in 16 index BC cases (detection rate among BC patients: 1.5%) and in 3 relatives affected by BC (Figure 2). On the other hand, *CHEK2* pathogenic or likely pathogenic germline variants were found in 16 index BC cases (detection rate among BC patients: 1.5%) and one relative affected by BC (Figure 2). The likely pathogenic and pathogenic variants of *ATM* and *CHEK2* detected in our population are listed in Table 2.

The final analysis included 36 BC patients (19 *ATM* mutation carriers and 17 *CHEK2* mutation carriers) affected by 46 different BCs, with ten patients developing bilateral BC. The characteristics of patients, tumors and treatments are outlined in Table 3.

Additionally, in 24 patients affected by both BC and OC, neither *ATM* nor *CHEK2* likely pathogenic or pathogenic variants were detected. In 76 OC patients, two *ATM* (one index case and one relative) and one *CHEK2* (index case) likely pathogenic or pathogenic variants were found. In 59 PC patients, finally, four *ATM* (all index cases) and one *CHEK2* (index case) likely pathogenic or pathogenic variants were detected.

### 3.2. ATM Mutation Carriers

Median age at first BC diagnosis in *ATM* mutation carriers was 46.9 years. Five patients (5/19, 26.3%) developed bilateral BC (one of them synchronous). Therefore, 24 tumors were analyzed in *ATM* carriers. Overall, 6 (25%) tumors were accounted for as ductal carcinoma in situ, while invasive ductal and lobular carcinoma amounted to 16 (66.7%) and 2 (8.3%), respectively. Invasive tumors were hormonal-receptor (HR) positive and HER2 negative (HR+/HER2–) in 9 (56.3%) cases, both HR and HER2 positive (HR+/HER2+) in 4 (25%) cases and triple negative in 3 (18.8%) cases. No HR–/HER2+ tumors were found. Thirteen (59.1%) early-stage BC (I/II stage) and three (13.6%) locally advanced tumors (III stage) were detected. No “de novo” metastatic BC were diagnosed.

Based on available data, seven (33.3%) tumors were treated by mastectomy and 13 (61.9%) through breast conserving surgery. One patient underwent axillary node dissection without breast surgery for CUP syndrome. Eleven (52.4%) sentinel node biopsies and four (19.1%) axillary node dissections were performed (with no axillary surgery in 6 cases). In 16 (84.2%) cases, radiation therapy followed breast surgery. Six out of 14 (42.9%) patients diagnosed with invasive BC underwent neoadjuvant chemotherapy, whereas 7 out of 13 (53.8%) patients underwent adjuvant chemotherapy. After a median follow up of 106 months, no local or distant recurrences were observed.

The most frequent mutation detected in the *ATM* gene was c.6154G>A, p.Glu2052Lys (5 out of 19 patients, 26.3%). Three of these women developed bilateral BC and five out of eight of these tumors were categorized as DCIS.

Twelve patients (63.2%) had a positive BC family history. In addition, a family history of ovarian, gastric, kidney/bladder and colon cancer were documented for 4 (21%) patients each, while a family history of pancreatic cancer was reported for 3 (15.8%) patients. One patient carrying an *ATM* pathogenic mutation with BC also developed gastric cancer. Moreover, two cases of epithelial ovarian cancer and four cases of pancreatic cancer were detected in six carriers of *ATM* pathogenic or likely pathogenic germline variants.

### 3.3. CHEK2 Mutation Carriers

Median age at first BC diagnosis in *CHEK2* mutation carriers was 46.1 years. Five patients (5/17, 29.4%) had bilateral BC (two of them synchronous). Therefore, 22 *CHEK2*-associated tumors were analyzed. Overall, 6 (30%) tumors were accounted for as ductal carcinoma in situ (Stage 0), while invasive ductal and lobular carcinoma amounted to 11 (55%) and 3 (15%), respectively. Invasive tumors were HR positive and HER2 negative (HR+/HER2–) in 11 (78.6%) cases, both HR and HER2 positive (HR+/HER2+) in 3 (21.4%) cases. No HR–/HER2+ and triple negative BCs were found. Eleven (55%) early-stage BC (I/II stage), one (5%) locally advanced tumor (III stage) and 2 (10%) stage IV cancers were diagnosed.

In patients diagnosed with localized BC, 10 (55.6%) tumors were treated by mastectomy and eight (44.4%) by breast conserving surgery. Seven (38.9%) sentinel node biopsies and seven (38.9%) axillary node dissections were performed (with no axillary surgery in 4 cases). In seven (41.2%) cases, radiation therapy followed breast surgery. One out of 11 (9.1%) patients diagnosed with invasive BC underwent neoadjuvant chemotherapy, whereas 4 out of 11 (36.4%) patients underwent adjuvant chemotherapy. After a median follow up of 152 months, only one local recurrence and no distant recurrences were observed in patients diagnosed with stage I–III BC.

The most frequent mutation detected in the *CHEK2* gene was c.190G>A, p.Glu64Lys (5 out of 17 patients, 29.4%). One of these women developed bilateral BC. The second most frequent pathogenic variant was c.470T>C, p.Ile157Thr and one case of bilateral tumor was observed in these women. The founder mutation c.1100delC, p.Thr367Metfs*15 was present in two patients.

Eleven patients (64.7%) had a positive BC family history and 6 (35.3%) patients one of colon cancer, whereas 4 (23.5%) patients had a prostate cancer family history and 4 (23.5%) patients one of kidney/bladder cancer. Six of our BC patients carrying a *CHEK2* pathogenic mutation were also diagnosed with thyroid carcinoma, acute myeloid leukemia, colon cancer, malignant melanoma, or uterine endometrial carcinoma. In addition to BC, the following malignancies were detected in five carriers of *CHEK2* pathogenic or likely pathogenic germline variants: pancreatic cancer, uterine cancer, prostate cancer, bladder cancer, multiple myeloma, kidney cancer, colon cancer, and thyroid carcinoma.

## 4. Discussion

The *ATM* and *CHEK2* genes encode proteins that act as tumor suppressors and are involved in the DNA damage response following generation of DNA double-strand breaks (DSBs) [4]. Second to the *BRCA1* and *BRCA2* genes, the most common germline pathogenic or likely pathogenic variants predisposing to BC were found in the *ATM* and *CHEK2* genes [2,3]. Individuals carrying heterozygous pathogenic variants in *ATM* or *CHEK2* present a 33% and 28–37% cumulative lifetime risk for BC by 80 years of age, respectively [7,8,9]. Nevertheless, while the phenotypes of BRCA-related tumors have been widely characterized, little is known about the clinicopathologic features of *ATM* and *CHEK2*-associated tumors, BC in the first place.

Interestingly, *CHEK2* has the highest mutation prevalence in individuals of European descent, while the spectrum and frequency of pathogenic variants vary among specific European populations. In particular, the frequency of the founder mutation c.1100delC declines from the north to the south of Europe, whereas the most frequent European *CHEK2* variant, p.I157T, has a carrier frequency of around 5% in Poles, Latvians, Hungarians and Russians and around 2–3% in Czechs, Slovaks and Germans [32]. In our study, the most frequent *CHEK2* mutation beyond c.470T>C, p.Ile157Thr (p.I157T), was c.190G>A, p.Glu64Lys. This likely pathogenic variant was observed at an allele frequency of 0.03% (38/126,668) in individuals of European ancestry in large population cohorts [33] and has been associated with a personal and/or family history of breast, prostate, ovarian, colorectal, thyroid and pancreatic cancer [34,35,36,37,38,39,40,41]. On the other hand, the most frequent *ATM* mutation in our population was c.6154G>A, p.Glu2052Lys. This likely pathogenic variant has also previously been reported in individuals with a personal and/or family history of breast and/or ovarian cancer [34,42]. It is noteworthy that one of the *ATM* mutations described in our population, c.2838+2162_4110-292del, has been recently characterized at a molecular level by our research group [43].

Overall, our study identified 19 *ATM* mutation carriers with 24 breast tumors and 17 *CHEK2* mutation carriers with 22 breast tumors. Median age at first BC onset was 46.9 years for *ATM* and 46.1 years for *CHEK2*, in keeping with the literature [44]. Moreover, a high rate of bilateral tumors was observed in *ATM* (26.3%) and *CHEK2* mutation carriers (41.2%). Previous studies differ from one another on the role of *ATM* mutations in increasing the risk of contralateral BC [45,46,47]. On the other hand, bilateral BC was reported for 3.7–12.1% of the patients harboring a *CHEK2* likely pathogenic or pathogenic variant [48,49], whereas a recent analysis has provided evidence of contralateral BC in 19.5% of Slovenian BC patients with *CHEK2* mutations [16]. Moreover, a systematic review and meta-analysis by Akdeniz et al. [50] has recently shown a strong association with contralateral BC for carriers of *CHEK2* c.1100delC mutation (relative risk, 2.7). In our study, however, only two patients with this variant were included (one of them with synchronous bilateral BC), so that no conclusion can be drawn. Interestingly, the low frequency of the founder mutation c.1100delC variant in our cohort of patients is consistent with a previous analysis reporting this variant in only 1 of 939 (0.11%, 95% CI = 0.00–0.59%) unrelated patients from Italian breast cancer families. These results indicate that the *CHEK2* c.1100delC variant has marginal relevance to breast cancer predisposition in the Italian population [51]. In our cohort, interestingly, all patients underwent genetic testing after breast surgery: this could have determined the high rate of contralateral BC since none of these women underwent risk-reducing contralateral mastectomy. Although the evidence on whether contralateral prophylactic mastectomy improves survival for *BRCA* carriers with BC is conflicting, this procedure reduces the risk of contralateral tumor by 93% [52]. As a result, several international guidelines include this option [10,53]. However, no studies have investigated risk reduction and survival advantage in relation to contralateral prophylactic mastectomy for patients with a diagnosis of BC harboring *ATM* or *CHEK2* likely pathogenic or pathogenic variants. In this regard, therefore, the decision should be shared with patients following a multidisciplinary and personalized approach.

In line with previous research [15,16,54,55,56,57] and unlike *BRCA1* and *PALB2*-associated BCs that commonly present triple-negative subtype [58,59], *ATM* and *CHEK2*-related BC in our population mostly resulted in luminal-like subtypes. In particular, 64.3% of the *CHEK2* tumors were luminal A-like, whereas most of the *ATM* tumors were luminal B-like/HER2-negative (56.2%). Interestingly, 81.2% of the *ATM* tumors and 100% of the *CHEK2* tumors were HR positive. In addition, 25% of *ATM* BCs and 21.4% of *CHEK2* BCs were observed to be HER2 positive, while only 18.8% of the *ATM* BCs and none of the the *CHEK2* tumors were accounted for as triple negative BC. Consistent with that, no *CHEK2* mutation carriers were observed in a previous analysis of 1824 triple negative breast cancer patients [60]. Contrary to what was described in previous experiences [61,62], it is notable that most of the *CHEK2* tumors (57.1%) were associated with lower grades (G1-G2). Finally, 21.4% of the *CHEK2*-related invasive tumors showed a lobular histotype, a high rate as previously highlighted in the literature [16,63,64]. In our population, however, due to the small sample size, the lobular histotype was not associated with any particular variant. On the other hand, *ATM* invasive BC in our population showed no particular histological subtype (88.9% were invasive ductal carcinomas and 11.1% were invasive lobular carcinomas), as already observed elsewhere [15].

About a quarter of all *ATM*-related BCs and a third of *CHEK2* BCs were in situ carcinomas and more than half of *ATM* and *CHEK2*-related BCs were diagnosed at stage I-II (59.1% and 55%, respectively), whereas 13.6% of the *ATM* BCs and 15% of the *CHEK2* BCs were stage III-IV. Despite the early stages at diagnosis, 55.6% of the *CHEK2* BCs were treated by mastectomy and 38.9% by axillary node dissection, since most of these patients underwent surgery in the late 90s when breast surgery was less conservative. A higher rate of *ATM*-associated BCs was treated with neoadjuvant chemotherapy (42.9%) and/or adjuvant chemotherapy (53.8%) compared to *CHEK2* tumors (9.1% and 36.4%, respectively), reflecting the higher percentage of stage III, HER2 positive and triple-negative BC in *ATM*-related tumors. Confirming good prognosis for luminal-like subtypes and early-stage BCs [65], after more than 8 years of follow up in both groups, only one local recurrence was observed in localized BCs at diagnosis.

As previously described for other cohorts [16,17], 63.2% of *ATM* mutation carriers and 64.7% of *CHEK2* mutation carriers presented a positive BC family history. Nevertheless, both germline *ATM* and *CHEK2* likely pathogenic or pathogenic variants have been linked with susceptibility to several malignancies other than BC. In our population, accordingly, *ATM* families exhibited ovarian cancer in 21% of cases and pancreatic cancer in 15.8% of cases, besides gastric, kidney/bladder and colon tumors. On the other hand, *CHEK2* families presented a recurrence of colon cancer (35.3% of cases), prostate tumors (23.5%) and kidney/bladder cancers (23.5%).

As has already been the case with BRCA-associated tumors, the definition of biological and clinical characteristics of *ATM* and *CHEK2*-related tumors may help improve diagnosis, prognostication and targeted therapeutic approaches [66,67,68]. In particular, in light of the high rate of contralateral tumors described in our experience, we believe that contralateral mastectomy should be considered and discussed with *ATM* and even more with *CHEK2* mutation carriers at the first diagnosis of BC. Further studies with larger patient cohorts are needed to confirm our findings and assist both patients and physicians in decision making and management recommendations in this subset of patients.

## Figures and Tables

**Table 1:** The MFCC criteria for genetic testing in BC, OC and PC cancer patients.

BC and OC in the Same Patient or Family.
OC, fallopian tube or primary peritoneal cancer (excluding mucinous and borderline) at any age.
Male BC
Triple negative BC diagnosed ≤60 years.
BC diagnosed ≤35 years.
PC at any age
At least two first-degree blood relatives with BC with at least one diagnosed ≤40 years or bilateral in the same family.

BC: breast cancer; OC: ovarian cancer; PC: pancreatic cancer.

**Table 2:** Likely pathogenic and pathogenic variants of *ATM* and *CHEK2* in our study population of BC patients.

Variants of ATM Detected	Variant Classification	Number of BC Patients	
c.6154G>A, p.Glu2052Lys	Likely pathogenic	5 index cases	1 relative
c.2838+2162_4110-292del	Pathogenic	1 index case	1 relative
c.5441T>A, p.Leu1814 *	Pathogenic	1 index case	1 relative
c.(8850+1_8851-1)_(*3591_?)del, p.(?)	Pathogenic	1 index case	-
c.8010+1delG	Likely pathogenic	1 index case	-
c.5697C>A, p.Cys1899 *	Pathogenic	1 index case	-
c.7327C>T, p.Arg2443 *	Pathogenic	1 index case	-
c.2192dupA, p.Tyr731 *	Pathogenic	1 index case	-
c.2135C>G, p.Ser712 *	Likely pathogenic	1 index case	-
c.8395_8404del10, p.Phe2799Lysfs *4	Pathogenic	1 index case	-
c.8814_8824del, p.Met2938Ilefs *	Pathogenic	1 index case	-
c.5932G>T, p.Glu1978 *	Pathogenic	1 index case	-
**Variants of CHEK2 detected**	**Variant classification**	**Number of BC patients**	
c.190G>A, p.Glu64Lys	Likely pathogenic	5 index cases	-
c.470T>C, p.Ile157Thr	Pathogenic	3 index cases	-
c.1169A>C, p.Tyr390Ser	Likely pathogenic	2 index cases	1 relative
c.1100delC, p.Thr367Metfs *15	Pathogenic	2 index cases	-
c.1189A>C, p.Tyr390Ser	Likely pathogenic	1 index case	-
c.592+3A>T, p.(?)	Likely pathogenic	1 index case	-
c.549G>C, p.Leu183Phe	Pathogenic	1 index case	-
c.85C>T, p.Gln29 *	Pathogenic	1 index case	-

**Table 3:** Patient and tumor characteristics.

	BC in *ATM* Mutation Carriers (*n* = 24)	BC in *CHEK2* Mutation Carriers (*n* = 22)
Median age at First BC Diagnosis, Years	46.9	46.1
Hystotype (n, %)	(*n* = 24)	(*n* = 22)
In situ ductal carcinoma	6 (25)	6 (30)
Invasive ductal carcinoma	16 (66.7)	11 (55)
Invasive lobular carcinoma	2 (8.3)	3 (15)
data not available	0	2
Clinical Stage at diagnosis (n, %)	(*n* = 24)	(*n* = 22)
is	6 (27.3)	6 (30)
I/II	13 (59.1)	11 (55)
III	3 (13.6)	1 (5)
IV	0	2 (10)
data not available	2	2
Immunohistochemical profile of invasive carcinomas (n, %)	(*n* = 18)	(*n* = 16)
HR+/HER2-	9 (56.3)	11 (78.6)
Luminal A-like	4 (25.1)	9 (64.3)
Luminal B-like	5 (31.2)	2 (14.3)
HR–/HER2+	0	0
HR+/HER2+	4 (25)	3 (21.4)
TNBC	3 (18.8)	0
data not available	2	2
Grade of invasive carcinomas (n, %)	(*n* = 18)	(*n* = 16)
G1-2	6 (42.8%)	8 (57.1%)
G3	8 (57.1%)	6 (42.8%)
data not available	4	2
Breast Surgery (n, %)	(*n* = 24)	(*n* = 22)
Mastectomy	7 (33.3)	10 (55.6)
Conserving surgery	13 (61.9)	8 (44.4)
No breast surgery	1 (4.8)	0
data not available or stage IV	3	4
Axillary Surgery (n, %)	(*n* = 24)	(*n* = 22)
Sentinel node biopsy	11 (52.4)	7 (38.9)
Axillary node dissection	4 (19)	7 (38.9)
No axillary surgery	6 (28.6)	4 (22.2)
data not available or stage IV	3	4
Radiotherapy (n, %)	(*n* = 24)	(*n* = 22)
Yes	16 (84.2)	7 (41.2)
No	3 (15.8)	10 (58.8)
data not available or stage IV	5	5
Neoadjuvant chemotherapy in invasive carcinomas (n, %)	(*n* = 18)	(*n* = 16)
Yes	6 (42.9)	1 (9.1)
No	8 (57.1)	10 (90.9)
data not available or stage IV	4	5
Adjuvant chemotherapy in invasive carcinomas (n, %)	(*n* = 18)	(*n* = 16)
Yes	7 (53.8)	4 (36,4)
No	6 (46.2)	7 (63,6)
data not available or stage IV	5	5
Local or distant recurrence in localized BC at diagnosis (n, %)	(*n* = 24)	(*n* = 20)
Yes	0 (0)	1 (5)
No	24 (100)	19 (95)
Median follow-up since diagnosis (months)	106	152

HR+/HER2-: hormonal-receptor positive and HER2 negative; HR–/HER2+: hormonal-receptor negative and HER2 positive; HR+/HER2+: hormonal-receptor and HER2 positive; TNBC: triple negative breast cancer.

## Data Availability

The data presented in this study are available within the article.
